# Evaluation of Aerosol Therapy during the Escalation of Care in a Model of Adult Cystic Fibrosis

**DOI:** 10.3390/antibiotics10050472

**Published:** 2021-04-21

**Authors:** Elena Fernández Fernández, Mary Joyce, Andrew O’Sullivan, Ronan MacLoughlin

**Affiliations:** 1 Medical Affairs, Aerogen Limited, Galway Business Park, H91 HE94 Galway, Ireland; efernandez@aerogen.com; 2 Research and Development, Science and Emerging Technologies, Aerogen Limited, Galway Business Park, H91 HE94 Galway, Ireland; mjoyce@aerogen.com (M.J.); aosullivan@aerogen.com (A.O.); 3 School of Pharmacy & Biomolecular Sciences, Royal College of Surgeons in Ireland, D02 YN77 Dublin, Ireland; 4 School of Pharmacy and Pharmaceutical Sciences, Trinity College, D02 PN40 Dublin, Ireland

**Keywords:** cystic fibrosis, lung disease, inhalation, nebuliser, aerosol therapy, antibiotic, escalation of care, home, hospital

## Abstract

Lung disease is the main cause of morbidity and mortality in cystic fibrosis (CF). CF patients inhale antibiotics regularly as treatment against persistent bacterial infections. The goal of this study was to investigate the effect of clinical intervention on aerosol therapy during the escalation of care using a bench model of adult CF. Droplet size analysis of selected antibiotics was completed in tandem with the delivered aerosol dose (% of total dose) assessments in simulations of various interventions providing oxygen supplementation or ventilatory support. Results highlight the variability of aerosolised dose delivery. In the homecare setting, the vibrating mesh nebuliser (VMN) delivered significantly more than the jet nebuliser (JN) (16.15 ± 0.86% versus 6.51 ± 2.15%). In the hospital setting, using VMN only, significant variability was seen across clinical interventions. In the emergency department, VMN plus mouthpiece (no supplemental oxygen) was seen to deliver (29.02 ± 1.41%) versus low flow nasal therapy (10 L per minute (LPM) oxygen) (1.81 ± 0.47%) and high flow nasal therapy (50 LPM oxygen) (3.36 ± 0.34%). In the ward/intensive care unit, non-invasive ventilation recorded 19.02 ± 0.28%, versus 22.64 ± 1.88% of the dose delivered during invasive mechanical ventilation. These results will have application in the design of intervention-appropriate aerosol therapy strategies and will be of use to researchers developing new therapeutics for application in cystic fibrosis and beyond.

## 1. Introduction

Cystic fibrosis (CF) is one of the most commonly diagnosed genetic diseases worldwide. It is caused by mutations in the cystic fibrosis transmembrane conductance regulator (CFTR) gene [1]. This encodes a cyclic AMP dependent chloride channel expressed in the epithelia of many exocrine tissues including the airways, lung, pancreas, liver, intestine, vas deferens and sweat gland/duct [2]. Mutations in CFTR cause abnormal ion transport in the epithelium of several tissues, which results in the production of abnormally thick and sticky mucus that blocks the organ and is responsible for CF pathology [3]. However, the main cause of morbidity and mortality in CF is lung disease with lung malfunction and pulmonary failure [4]. The production of sticky mucus in the lumen of the lungs impedes mucociliary clearance [5]. Clinically, this manifests as chronic inflammation and recurrent bacterial infections by pathogens such as *Pseudomonas aeruginosa* and *Staphylococus aureus* [6]. Mucus and inflammatory cells cause bronchiectasis, and the continuous cycle of infection and inflammation lead to the progressive destruction of the lung tissue [7,8]. Survival of patients with CF has continued to improve to a median age of 46.2 years. This has resulted in a rapid increase in the adult CF population and complications arising from the disease are becoming more common [9]. Therefore, pulmonary disease, one of the major factors linked to mortality [10], is a crucial point in the optimal management of the patients. Consequently, CF patients inhale antibiotics and other therapeutics regularly as treatment against those persistent bacterial infections. Daily treatment with mucolytics, DNase, antibiotics, bronchodilators (considered the “core treatments”), as well as corticosteroids, hypertonic saline and amilorides have been used in the treatment of CF lung disease [11]. As an example, adults with CF who are colonised with *Pseudomonas* will typically be prescribed at least twice daily nebulised antibiotics in addition to a once daily nebulised mucolytic [12]. The inhaled route offers the potential for high drug concentrations delivered directly to the target organ (airways and lung), eliminating the side effects of the systemic delivery route [13]. Thus, nebuliser performance is a key factor when optimising the delivery of inhaled medications.

**Figure 1:**
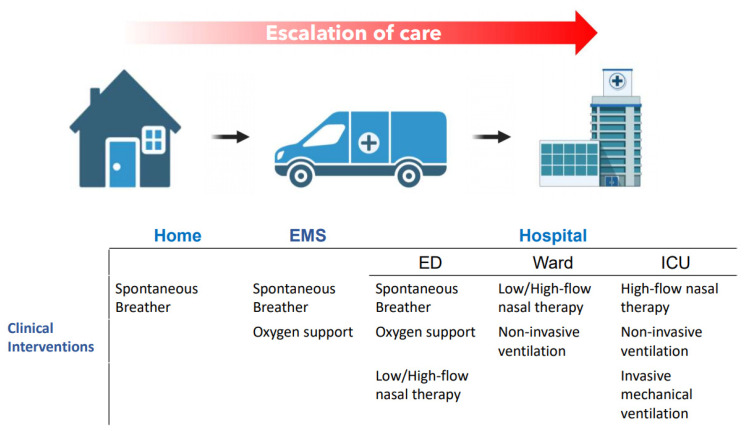
Schematic illustration of the potential escalation of care for adult CF patients and the clinical interventions commonly used in each of the different care settings.

The treatment burden is so high in these CF patients that the optimisation of the delivery device may assist individuals in completing the prescribed treatment regimes in different care settings, such as at home or when requiring further treatment during escalation of care. For example, in the case of an acute pulmonary exacerbation, when the patient is admitted in the hospital [14]. The escalation of respiratory support with concurrent aerosol therapy for CF patients likely plays a role in the success of clinical outcomes. These respiratory supports act to deliver supplemental oxygen to the patient, for example, low flow nasal therapy (LFNT) or high flow nasal therapy (HFNT), or provide ventilatory support, for example, non-invasive mechanical ventilation (NIV) or invasive mechanical ventilation (IMV). Figure 1 illustrates the potential escalation of care of CF patients, from home to the hospital setting, and their respiratory support upon the site of care. During that escalation of care, concurrent aerosol therapy may be performed, and the nebulisers used by CF patients may differ within the different scenarios.

Of the two main types of nebulisers, compressed-air-driven jet nebulisers (JN) are the most common in the home. Vibrating mesh nebuliser-type (VMN) is also used in the home, but to a lesser degree. Whilst JN is available in the hospital setting, the VMN is the predominant type. VMN is the only one considered suitable for concurrent use across the various patient interventions encountered throughout the escalation of care from home to intensive care [15,16]. Additionally, current global COVID−19 guidance on the selection of nebuliser recommends the use of devices that maintain a closed system for the prevention of release of both medical and patient-derived bioaerosols into the local environment. VMN is the only nebuliser type that allow this [17,18,19,20,21].

The aim of this study was to investigate the effect of the escalation of care related to the aerosol therapy delivered to the patient under a simulated adult CF breathing profile, and to provide insight into the variability of nebulised dose delivery across clinical interventions either in the homecare or hospital setting.

## 2. Results

### 2.1. Respirable Fraction Aerosol Droplet Size Characterisation

The result of droplet size characterisation for a selection of commonly nebulised antibiotic formulations using the Aerogen Solo vibrating mesh nebuliser are presented in Table 1 below. All formulations characterised were within the respirable range with relatively high fine respirable fractions.

### 2.2. Simulated Aerosol Delivery during Spontaneous Breathing in the Homecare Setting

**Figure 2:**
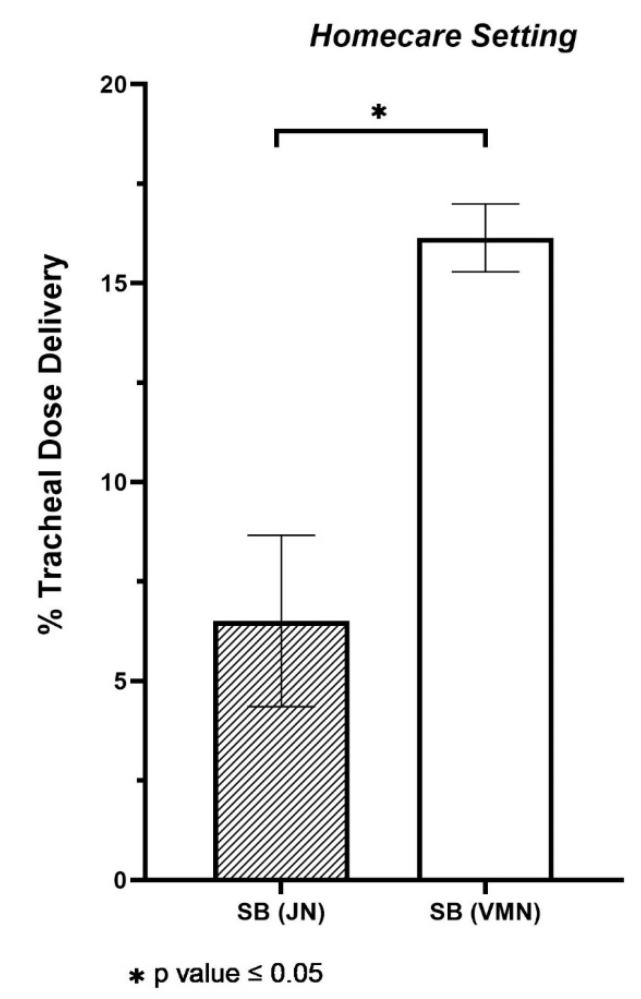
Aerosol drug delivery to the level of the trachea with two nebuliser types e.g., vibrating mesh nebuliser (VMN) in combination with a mouthpiece and no supplemental flow and jet nebuliser (JN) with open facemask at 7 L per minute compressed air flow rate during spontaneous breathing (SB). The values represented are the mean averages ± SD of five independent experiments. Differences were considered statistically significant when * *p* ≤ 0.05.

Following tests, the JN was seen to deliver 6.51 ± 2.15% of the nominal dose to the end of the trachea, i.e., the expected lung dose. However, the portable VMN was seen to deliver significantly more, with 16.15 ± 0.86% of the dose delivered to the end of the trachea (*p* value < 0.001). See Figure 2. The consequence of device selection is apparent here with choice of nebuliser having a significant bearing on the amount of drug delivered to the patient.

### 2.3. Simulated Aerosol Delivery in the Hospital Setting

#### 2.3.1. Emergency Department

**Figure 3:**
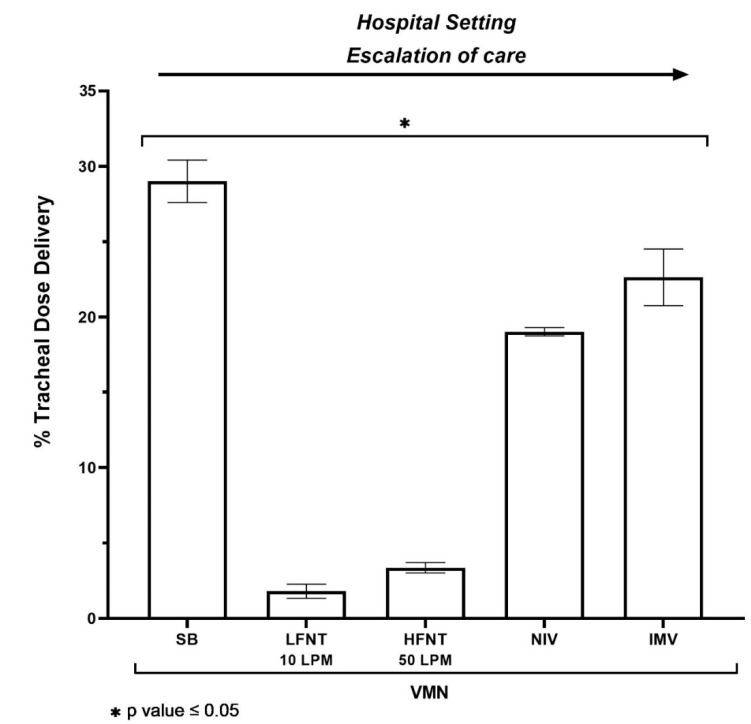
Aerosol drug delivery to the level of the trachea using a vibrating mesh nebuliser (VMN) across several combinations of clinical intervention e.g., spontaneous breathing (SB) in combination with the VMN, mouthpiece and no supplemental flow, low flow nasal therapy (LFNT) at 10 LPM, high flow nasal therapy (HFNT) at 50 LPM, non-invasive ventilation (NIV) and invasive mechanical ventilation (IMV). The values represented are the mean averages ± SD of five independent experiments. Differences were considered statistically significant when * *p* ≤ 0.05.

In simulated aerosol drug delivery interventions and oxygen therapy interventions that facilitate concurrent aerosol drug delivery commonly prescribed for patients presenting to the hospital emergency department, mouthpiece-mediated VMN aerosol delivery with no supplemental gas flow rate was seen to deliver the highest dose, with 29.02 ± 1.41% of the nominal dose delivered to the level of the trachea (*p* value < 0.001 versus low flow and high flow systems, respectively). When the same VMN device was included in a low flow nasal therapy system at 10 L per minute, 1.81 ± 0.47% of the dose was delivered, compared with 3.36 ± 0.34% delivered during high flow nasal therapy at 50 L per minute oxygen flow rate. See Figure 3. In this instance, whilst the same nebuliser type was used, it is obvious that clinical intervention can have a significant bearing on the amount of drug delivered to the patient.

#### 2.3.2. Ward/Intensive Care Unit

During both non-invasive and invasive mechanical ventilation, typical of the intensive care and ward settings, similar levels of drug were seen delivered under ventilation strategies appropriate for the adult cystic fibrosis patient. NIV in combination with VMN recorded 19.02 ± 0.28%, whereas IMV recorded 22.64 ± 1.88% of the dose delivered (*p* value = 0.012). See Figure 3.

## 3. Discussion

Aerosol therapy is a mainstay in the routine treatment of CF wherein many therapeutics, including antibiotics, are delivered via nebuliser. Here, we have assessed aerosol drug delivery across a range of CF-appropriate interventions in both the homecare and hospital settings. Using a tracer drug, salbutamol, in combination with adult CF breath patterns, we describe the variability in dose delivery that could be expected to be delivered to the CF patient. This study, for the first time, investigates the effect of device and intervention on the amount of drug delivered to the patient throughout the escalation of care of a simulated exacerbated adult CF patient.

Droplet size is known to affect aerosol drug delivery in the patient, and here we characterised the respirable fraction and droplet size of a selection of commonly nebulised antibiotics [22]. Using a single nebuliser type as the control, indicative droplet size was seen to be relatively consistent with all formulations under test, recording a consistent respirable fraction and droplet size. This would indicate that whilst some differences are noted between formulations, they all remain within the respirable range. Factors affecting droplet size include the physicochemical characteristics, drug concentration, ionic content and temperature of the formulation being aerosolised [23,24,25,26]. Further, significant variation in aerosol characteristics can be expected between devices [27,28]. As such, there will never be a single droplet size distribution that can be relied on, and so these results should be considered as only a feasibility for generation of inhalable antibiotic.

In the home, the predominant nebuliser type used is the JN [29]. In more recent times, however, the vibrating mesh type devices have seen increasing adoption [30,31,32]. These VMN devices claim higher drug delivery rates, with less drug wastage. Here, the results demonstrate that, in this case at least, the portable VMN delivered approximately 2.5 times more to the simulated patient than the JN. The drug/mass amount delivered, whilst not described in the literature previously using the breathing pattern used herein, are in line with results published in imaging studies assessing performance of the JN in a spontaneously breathing adult patient (for example, 6.51% versus 5.2%) [31]. Whilst only one JN type was assessed here, variability in JN performance can be significant, and so these results should be considered representative of the nebuliser type [32]. The dramatic difference in tracheal dose delivered is due to a combination of factors including the large residual volume recorded in JN of all types [33,34], as well as the fact that the drug mass tends to concentrate in the residual volume remaining, as buffer is preferentially aerosolised [23,35].

Should the patient require medical attention and present at the emergency department (ED) in the hospital, a variety of patient interventions may be prescribed in an effort to have the patient return to stable-state lung function. On confirmation of infection, or infection-mediated bronchospasm, inhaled antibiotics, mucolytics or bronchodilators may be prescribed [36,37]. Whereas JN are found and used in the ED, VMN is also, with a mounting body of published clinical literature supporting its adoption [38,39,40]. As mentioned, VMN is also considered the only nebuliser type suitable for concurrent use with a variety of oxygenation supplementation or ventilatory support strategies. In fact, in some cases, JN is contra-indicated against, as the compressed air required for its normal function interferes with the finely tittered oxygen flow prescribed for the patient [41]. In the ED setting, and depending on how severe the state of the patient, and their supplemental oxygen requirement, e.g., if they become hypoxic, they may be prescribed further aerosol therapy. This may be concurrent with low flow nasal therapy (LFNT) or high flow nasal therapy (HFNT). Across these frontline interventions, our studies indicate that aerosol delivery performance can vary significantly. As might be expected, an interface such as the mouthpiece-mediated delivery using the VMN and chamber was seen to deliver the greatest amount of tracer aerosol during simulated breathing, with LFNT and HFNT delivering significantly less. The results recorded here for VMN-mediated aerosol delivery are in line with previous reports for the VMN in combination with the chamber (for example, 29.02% versus 34.1%) [31,42], and HFNT (for example, 3.36% versus 3.46%) [42,43]. No experimentally comparable published data for LFNT could be found.

The larger dose delivered by the combination of VMN and chamber, via mouthpiece, may be explained through a combination of bolus aerosol inhalation, whereby aerosol builds up in the chamber during the exhalation phase of the breath, and the relatively large bore oropharynx, which minimises losses before the lung. The HFNT result is explained through significant aerosol losses throughout the tubing between the nebuliser and the patient but, additionally, the impaction losses seen in the nasal passages after the aerosol exits the nasal cannula [44]. One would expect that, at the lower oxygen flow rates used during LFNT, the ballistic fraction would be smaller and, thus, impaction losses would be minimised; however, a significant fraction of the aerosol is lost in the nasal passages [45]. Further, a major contributor to the reduced aerosol dose here is the very narrow bore oxygen cannula that are typically used [44]. In this instance, the inner diameter of the cannula was 1.8 mm. This is low, compared with the typical internal diameter of adult HFNT cannula, measuring up to as wide as 10 mm [44]. The ratio of nasal cannula outer diameter to nostril inner diameter determines the leak of oxygen, and consequently aerosol from the system also. This ratio also plays a part in the delivered aerosol dose, as well as the levels of fugitive emissions to the local environment, which is a key, but often forgotten consideration for healthcare professionals or bystanders during aerosol therapy [46,47,48,49]. A recent review of aerosol delivery during HFNT by Li and colleagues provides additional insight into the factors at play [50].

Clinically, should these oxygenation supplementation strategies not be successful, and the patient continues to deteriorate, non-invasive ventilation (NIV), or as a last resort, invasive mechanical ventilation (IMV), may be prescribed. These ventilatory support strategies provide pre-set minimum pressures and/or volumes of air and aid the patient in returning to normal pulmonary arterial oxygen levels. NIV is often deployed as pressure support in a conscious spontaneously breathing patient, with IMV having a selection of ventilatory modes available for ventilation of the anaesthetised, unconscious patient via an endotracheal tube. In this study, there were only small differences between NIV and IMV with respect to the delivered aerosol dose (19.02% versus 22.64% respectively). Again, these figures are in line with previous published reports [51,52].

Whilst little difference was noted between NIV and IMV, these interventions deliver significantly more aerosol compared to LFNT and HFNT. The likely reasons for the poor delivery during those interventions has already been explained; however, NIV benefits from large bore tubing (22 mm inner diameter) and a tight-fitting facemask (no leak). Additionally, with the addition of a fixed minimum positive end expiratory pressure (PEEP), aerosol distribution and deposition is likely to be increased over the lower PEEP levels delivered by LFNT and HFNT [53]. IMV also benefits from larger bore tubing, and depending on the ventilator mode, the bias flow-mediated build-up of an aerosol bolus between inhalations [54]. Furthermore, and to ensure safe volumes and pressures are delivered to the patient’s lung on each breath, air leak is minimal, with cuffed endotracheal tubes creating a complete seal within the trachea. This again not only facilitates increased aerosol delivery, but also reduces fugitive medical aerosol emissions to the local environment, and, cognizant of COVID−19, the risk of patient derived transmission of infectious disease [55,56].

The implications of these findings may be far-reaching. For example, patients need to be aware of the potential differences between nebulisers when purchasing from a community pharmacy; however, in some instances this choice may be limited if a particular nebuliser is indicated for use on the drug label. Further, clinicians now have insight into potential variability in aerosol delivery across common clinical interventions, and may, where appropriate, titrate the dose accordingly. Finally, developers of novel therapeutics for the treatment of CF are now provided insight into the significant variance in delivery between devices and interventions, and should consider the intended patient and treatment carefully in the selection of devices and interventions for delivery of their therapeutic.

## 4. Materials and Methods

### 4.1. Nebulisers

Experiments were conducted using a selection of appropriate and commonly used nebulisers in the treatment of cystic fibrosis, and the escalating treatment of lung infection. In the homecare setting, both a compressed air-driven jet nebuliser (Up-draft II jet nebuliser, Hudson, Teleflex Medical, Wake County, NC, USA) as well as a portable vibrating mesh nebuliser (InnoSpire Go, Philips, Farnborough, UK) were evaluated. In the hospital setting, a second VMN was used (Aerogen Solo, Aerogen Ltd., Galway, Ireland).

### 4.2. Respirable Fraction Aerosol Droplet Size Characterisation

The respirable fraction and aerosol droplet size for a selection of antibiotic formulations were characterised using laser diffraction as previously described [57]. Briefly, the devices were loaded with sample and connected to the inlet of the droplet sizer (Spraytec, Malvern Instruments, Worcestershire, UK). The nebuliser was turned on and run until the entire dose was delivered. Testing was carried out in triplicate. The aerosol characteristics of the nebulisers, with respect to volumetric median diameter (VMD) and fine particle fraction (FPF), were measured using laser diffraction (Spraytec, Malvern Instruments, Worcestershire, UK).

### 4.3. Determination of Tracheal Dose

For all test combinations, 2.5 mL of 2.5 mg/2.5 mL of Albuterol Sulphate (Ventolin, GlaxoSmithKline, Brentford, UK) was used as a tracer aerosol drug to determine drug dose delivered. This is in line with previous studies [44,54,58,59] and international nebuliser test standard ISO27427. A 3D printed adult head model [60,61] was attached to a breathing simulator (BRS2100, Copley Scientific Ltd., Nottingham, UK) via a capture filter, at the level of the trachea bifurcation (Respirgard 303, Vyaire, Basingstoke, UK) and breathing pattern applied. See Table 2 for summary of simulated aerosol delivery test scenarios with associated nebuliser types, interfaces and breath patterns utilised for each test. At the end of each dose, the drug captured on the filter was eluted using 10 mL of deionised water. The mass of drug was quantified by means of UV spectrophotometry at a wavelength of 276 nm and interpolation on a standard curve of albuterol sulphate concentrations (100–3.125 µg/mL). Results for tracheal dose were expressed as the percentage of the nominal dose placed in the nebuliser’s medication cup. All testing was carried out in independent quintuplicate.

### 4.4. Simulated Aerosol Delivery during Spontaneous Breathing

**Figure 4:**
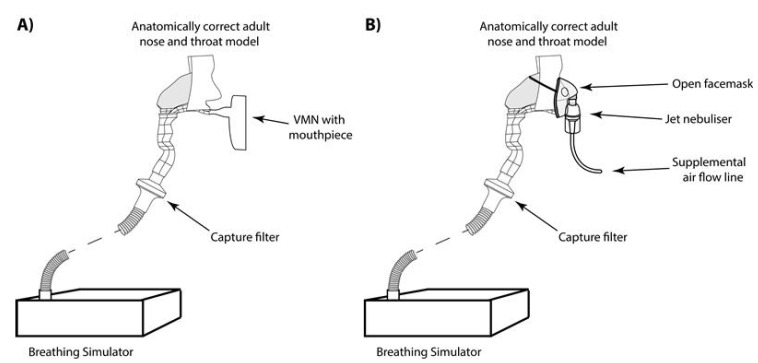
Portable VMN in combination with a mouthpiece (**A**) and JN plus open facemask (**B**).

To replicate aerosol therapy in a spontaneously breathing adult CF patient undergoing standard care in the homecare setting, both the JN plus open facemask (Up-draft II jet nebuliser, Hudson, Teleflex Medical, Wake County, NC, USA) and portable VMN in combination with a mouthpiece (InnoSpire Go, Philips, Farnborough, UK) were used. The JN was operated at 7 L per minute compressed air flow rate, in line with the accompanying instruction manual. To replicate aerosol therapy in a spontaneously breathing adult CF patient post-exacerbation in the hospital setting a VMN in combination with an aerosol chamber and filtered mouthpiece (Aerogen Ultra, Aerogen Limited, Galway, Ireland) (Respirgard 303 filter, Baxter, Dublin, Ireland) with no supplemental oxygen was used to deliver drug to the simulated adult CF patient. See Figure 4A,B.

### 4.5. Simulated Aerosol Delivery during A Non-Invasive Patient Intervention–Nasal Oxygen Support

**Figure 5:**
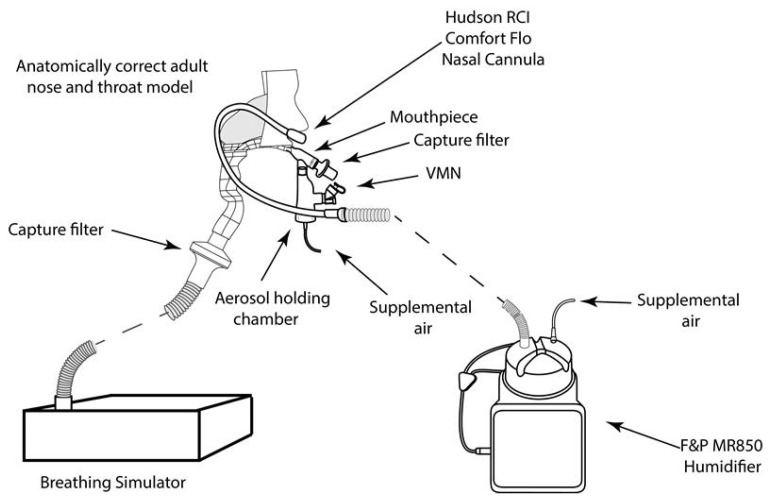
LFNT setup, the VMN (Aerogen Ultra, Aerogen Limited, Galway, Ireland) was used with a mouthpiece and, concurrently, nasal oxygen cannula placed in the nostrils of the head model.

Low flow oxygen therapy (LFNT): A nasal oxygen cannula (Hudson RCI comfort flo^®^ nasal cannula, Teleflex Medical, Delaware County PA, USA) placed in the nostrils of the head model was used to simultaneously deliver low flow oxygen therapy at 10 LPM while aerosol from the VMN (Aerogen Solo, Aerogen Limited, Galway, Ireland) was delivered in combination with an aerosol chamber and filtered mouthpiece (Aerogen Ultra, Aerogen Limited, Galway, Ireland) (Respirgard 303 filter, Vyaire, Basingstoke, UK), with no supplemental oxygen. See Figure 5.

**Figure 6:**
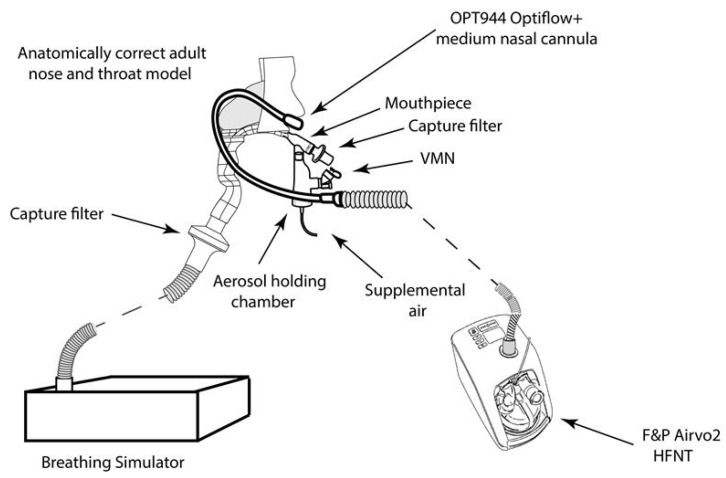
HFNT setup, the VMN (Aerogen Ultra Aerogen Limited, Galway, Ireland) was used with a mouthpiece and nasal cannula placed in the nostrils of the head model.

High flow oxygen therapy (HFNT): A nasal cannula (OPT944, Optiflow + Medium, Fisher & Paykel, New Zealand) placed on the head model was used to deliver oxygen at 50 LPM with the turbine driven HFNT device (AIRVO2, Fisher & Paykel, Auckland New Zealand). Aerosol from the VMN (Aerogen Solo, Aerogen Limited, Ireland) was delivered in combination with an aerosol chamber and filtered mouthpiece (Aerogen Ultra, Aerogen Limited, Galway, Ireland) (Respirgard 303 filter, Vyaire, Basingstoke UK), with no supplemental oxygen. See Figure 6.

### 4.6. Simulated Aerosol Delivery during Mask-Mediated Non-Invasive Ventilation (NIV)

**Figure 7:**
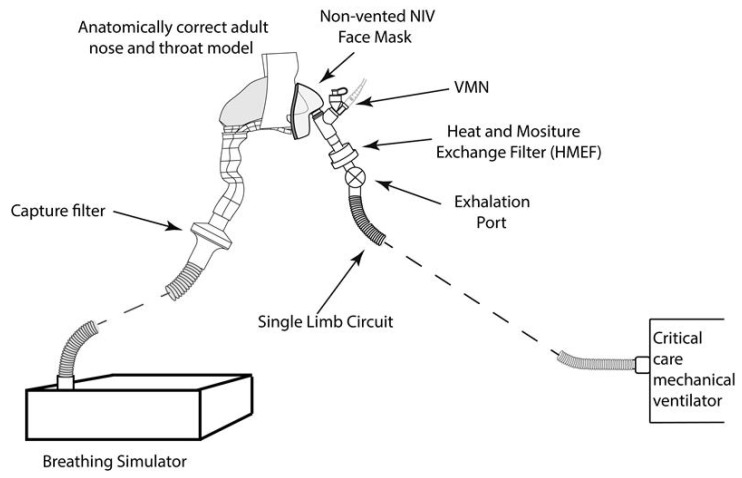
NIV setup, a critical care mechanical ventilator with non-invasive functionality and the VMN placed between the exhalation port of the single limb circuit and the mask.

A critical care mechanical ventilator with non-invasive functionality (Bellavista, IMT Medical, Buchs Switzerland), was set to deliver continuous positive airway pressure (CPAP) of 10 cmH20. It was attached to the breathing simulator via a single limb, vented circuit with a non-vented NIV mask (RT045, Nivairo, Fisher and Paykel, Auckland New Zealand). The breathing simulator was set to deliver the same breathing pattern as the spontaneously breathing patient (see Table 2). In line with published in vivo imaging studies of the optimal nebuliser placement, the VMN was placed between the exhalation port of the single limb circuit and the mask [65]. The mask was attached to the breathing simulator via a capture filter. See Figure 7.

### 4.7. Simulated Aerosol Delivery during Endotracheal Tube-Mediated Invasive Mechanical Ventilation (IMV)

**Figure 8:**
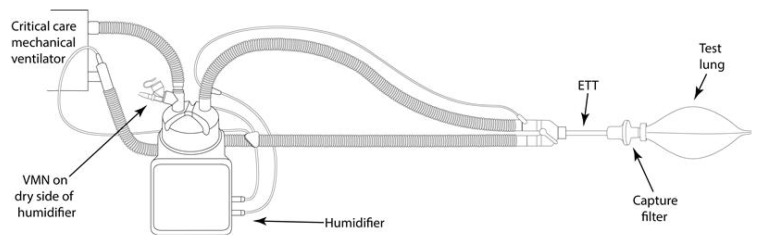
IMV setup, the VMN was placed on the dry side of a heated humidifier and the capture filter placed between the ETT and a test lung.

A critical care mechanical ventilator (Servo-U, Maquet, Baden-Württemberg, Germany) incorporating a dual limb circuit (RT200, Fisher & Paykel, Auckland New Zealand) was used in combination with a humidifier (MR850, Fisher & Paykel, Auckland New Zealand). The VMN was placed at the dry side of the humidifier. A lung protective, low tidal volume ventilation strategy of 8 mL/Kg for a 69 Kg adult was adopted (Vt 550 mL, RR 13 BPM, and I:E Ratio 1:2) [64]. The capture filter was placed between the endotracheal tube (ETT) (Flexicare, 8.00 mm, UK) and a test lung (IMT Medical, Bachs Switzerland). See Figure 8.

### 4.8. Statistical Analysis

Data are expressed as the arithmetic mean ± standard deviation (SD) tracheal dose (percentage). Statistical analysis was carried out using Minitab Software, version 18 (Coventry, UK). Student’s t-tests were conducted to establish if the tracheal dose varied significantly across two different aerosol generators in the homecare setting. A One-Way ANOVA was performed to assess different drug delivery interfaces, across various patient interventions using a VMN only, in the hospital setting. Differences were considered statistically significant when *p* ≤ 0.05 (*). Aerosol droplet size characterisation work was carried out in triplicate. Tracheal dose experiments were conducted in quintuplicate and with at least five technical replicates per experiment.

## 5. Study Limitations

The experimental approach taken here to describe the gross or total aerosol inhaled and delivered to the level of the trachea does not consider the disease specific features of CF such as mucus secretions, inflammation, infection etcetera and the influence they may have on distribution and deposition throughout the airways.

## 6. Conclusions

This study highlights the potential for sometimes significant variance in aerosol drug delivery between both nebuliser type, and clinical intervention. These results will have application in the design of intervention-appropriate aerosol therapy strategies and will be of use to researchers developing new therapeutics for application in cystic fibrosis and beyond, as well as clinicians in the selection of nebuliser/intervention combinations in the treatment of patients.

## Figures and Tables

**Table 1:** Results of aerosol droplet size analysis indicative for commonly nebulised antibiotic formulations using the Aerogen Solo vibrating mesh nebuliser. The values represented are mean ± standard deviation (expressed in percentage) of three independent experiments.

Formulation	Concentration	Manufacturer	VMD (μm)	Flow Rate (mL/min)	FPF (%) <5 μm	FPF (%) <3 μm	FPF (%) 1–5 μm
Saline	0.9%	BBraun, Ireland	4.43 ± 0.01	0.45 ± 0.01	57.45 ± 0.08	28.59 ± 0.07	56.05 ± 0.03
Salbutamol	2.5 mg/2.5 mL	GlaxoSmithKline	4.07 ± 0.43	0.46 ± 0.01	61.38 ± 0.38	34.66 ± 0.25	56.11 ± 0.59
Genticin	80 mg/2 mL	Amdipharm	4.21 ± 0.01	0.48 ± 0.01	59.77 ± 0.07	32.49 ± 0.21	55.87 ± 0.34
Tobramycin	80 mg/2 mL	Hospira	4.25 ± 0.01	0.47 ± 0.01	59.28 ± 0.10	31.85 ± 0.11	56.15 ± 0.27
Likacin	500 mg/2 mL	TitoLare	4.78 ± 0.01	0.51 ± 0.01	52.08 ± 0.08	31.25 ± 0.04	43.11 ± 0.13
Colistin	0.25 million IU/4 mL	Teva	4.05 ± 0.03	0.21 ± 0.01	57.97 ± 0.43	39.45 ± 0.51	44.97 ± 0.53

**Table 2:** Summary of study combinations with associated nebuliser types, interfaces and breath patterns utilised for each test. (LFNT = Low Flow Nasal Therapy; HFNT = High Flow Nasal Therapy; Vt = tidal volume; RR = respiratory rate; BPM = Breath per minute; I:E = Inspiratory: Expiratory ratio; LPM = Litres per minute).

Simulated Aerosol Delivery Test Scenario	Nebuliser Type	Interface	Simulated Breath Parameters
Spontaneous breathing (SB)	VMN	Mouthpiece	Vt 410 mL RR 22 BPM I:E Ratio 1:2 [62,63]
	JN	Open facemask	
Non-invasive patient intervention nasal oxygen	VMN	Nasal cannula with LFNT at 10 LPM	
	VMN	Nasal cannula with HFNT at 60 LPM	
Non-invasive ventilation (NIV)	VMN	Non-vented NIV mask	
Invasive mechanical ventilation (IMV)	VMN	Endotracheal tube	Vt 550 mL RR 13 BPM I:E Ratio 1:2 [64]

## Data Availability

All relevant data is contained within the article.
